# Transient Decrease in Circulatory Testosterone and Homocysteine Precedes the Development of Metabolic Syndrome Features in Fructose-Fed Sprague Dawley Rats

**DOI:** 10.1155/2016/7510840

**Published:** 2016-10-12

**Authors:** Anil Sakamuri, Sujatha Pitla, Uday Kumar Putcha, Sugeedha Jayapal, Sailaja Pothana, Sai Santosh Vadakattu, Nagabhushan Reddy Konapalli, Siva Sankara Vara Prasad Sakamuri, Ahamed Ibrahim

**Affiliations:** ^1^Department of Biochemistry, National Institute of Nutrition, Indian Council of Medical Research, Jamai Osmania PO, Hyderabad, Andhra Pradesh 500 604, India; ^2^Department of Pathology, National Institute of Nutrition, Indian Council of Medical Research, Jamai Osmania PO, Hyderabad, Andhra Pradesh 500 604, India; ^3^National Center for Laboratory Animal Sciences, Indian Council of Medical Research, Jamai Osmania PO, Hyderabad, Andhra Pradesh 500 604, India

## Abstract

*Background*. Increased fructose consumption is linked to the development of metabolic syndrome (MS). Here we investigated the time course of development of MS features in high-fructose-fed Sprague Dawley rats along with circulatory testosterone and homocysteine levels.* Methods*. Rats were divided into control and experimental groups and fed with diets containing 54.5% starch and fructose, respectively, for 4, 12, and 24 weeks. Plasma testosterone and homocysteine levels were measured along with insulin, glucose, and lipids. Body composition, insulin resistance, and hepatic lipids were measured.* Results*. Increase in hepatic triglyceride content was first observed in metabolic disturbance followed by hypertriglyceridemia and systemic insulin resistance in fructose-fed rats. Hepatic lipids were increased in time-dependent manner by fructose-feeding starting from 4 weeks, but circulatory triglyceride levels were increased after 12 weeks. Fasting insulin and Homeostatis Model Assessment of Insulin Resistance (HOMA-IR) were increased after 12 weeks of fructose-feeding. Decreased visceral adiposity, circulatory testosterone, and homocysteine levels were observed after 4 weeks of fructose-feeding, which were normalized at 12 and 24 weeks.* Conclusions*. We conclude that transient decrease in circulatory testosterone and homocysteine levels and increased hepatic triglyceride content are the earliest metabolic disturbances that preceded hypertriglyceridemia and insulin resistance in fructose-fed SD rats.

## 1. Background

Cardiovascular diseases (CVDs) and type 2 diabetes are the major causes of morbidity and mortality in developed and developing countries [1]. Evidences from epidemiological studies have suggested that metabolic syndrome is a leading cause of CVDs and type 2 diabetes [2, 3]. MS is a complex pathophysiological entity characterized by insulin resistance (IR), central obesity, hypertension, and dyslipidemia [4]. Both genetic factors and environmental factors like sedentary lifestyle and high calorie intake result in MS development [4]. Recent studies reported that high fructose consumption is an important contributing factor which onsets MS [5]. In human subjects, fructose consumption is positively associated with dyslipidemia, insulin resistance, and obesity [6, 7]. These observations are further supported by animal studies, where feeding of fructose-rich diets resulted in all features of MS [8]. High-fructose-fed rat model is a well-established animal model to elucidate mechanisms involved in fructose-induced MS development and also to study the effect of various nutrient and drug interventions on MS [8]. Majority of the studies on fructose-induced MS are single time point studies resulting in more than one MS feature [8]. As each of the MS features significantly increases the risk of the other, time course studies are needed to better understand the sequential development of MS risk factors. These studies are expected to help in designing experiments to elucidate the mechanisms involved in the development of individual feature of fructose-induced MS and to study the influence of each risk factor on others and also to study effect of therapeutic drugs on different MS risk factors.

Male reproductive hormone, testosterone, also regulates various cellular and metabolic functions that are linked to development of MS. Components of MS including hypertriglyceridemia, nonalcoholic fatty liver disease, IR, and visceral obesity are shown to be associated with low testosterone levels in human subjects [9–11]. Several studies showed that low testosterone levels are associated with MS and increase the risk of obesity, type 2 diabetes, and CVDs [9–11]. In support to these observations, testosterone replacement therapy in men improved MS features [12]. Similar observations were also reported in animal models, but studies reporting testosterone levels during the course of development of fructose-induced MS have not been reported.

Homocysteine, a nonprotein amino acid formed during conversion of methionine to cysteine, was shown to be a critical determinant of CVD risk [13]. Both clinical and animal studies showed association of plasma homocysteine levels with fatty liver, dyslipidemia, obesity, and insulin resistance [14–16]. Homocysteine levels were reported to be higher in MS subjects [17]. Very few studies reported circulatory homocysteine levels in fructose-induced MS animal models [18].

In the present study, we investigated the sequential development of MS risk factors including fatty liver, insulin resistance, dyslipidemia, and visceral adiposity by feeding high-fructose diet (54.5%) for different durations to Sprague Dawley rats, one of the extensively used rat strain for fructose-induced MS experiments. Another main objective of our study is to determine the circulatory testosterone and homocysteine levels during the course of MS development.

## 2. Methods

### 2.1. Animal Experiment

Weanling male, Sprague Dawley rats (*n* = 48) were obtained from National Center for Laboratory Animal Sciences, Hyderabad, India, and divided equally into control and experimental groups. Control rats were fed with diet containing starch (54.5%) as carbohydrate source, whereas rats in experimental group were fed with fructose (54.5%) for 4-week, 12-week, and 24-week durations (*n* = 8). Diet compositions were similar across the groups except for carbohydrate source and all the diets were prepared as per AIN-93 recommendations. Daily food intake and weekly bodyweights were recorded. At the end of each time point, rats (*n* = 8/group) were sacrificed after overnight fast by CO_2_ asphyxiation and blood plasma was collected. Tissues were collected and stored at −80°C for further analysis. All protocols were approved by Institutional Animal Ethical Committee (IAEC).

### 2.2. Plasma Total Testosterone and Homocysteine Analysis

Plasma total testosterone levels were measured by kit based method using ELISA (Labor Diagnostika Nord GmbH & Co., KG, Germany). Plasma homocysteine levels were measured by HPLC using a SUPELCOSIL™ LC-18-DB (150 mm by 4.6 mm) column according to methods reported previously [19].

### 2.3. Plasma and Hepatic Lipid Analysis

Plasma triglycerides and total and HDL cholesterol were measured by enzyme-based kit (Biosystems, Barcelona, Spain) methods. Hepatic lipids were extracted by Folch's method [20] and total triglycerides were quantified by kit (Biosystems, Barcelona, Spain) method as described above.

### 2.4. Oral Glucose Tolerance Test

After overnight fasting, a zero-hour blood sample was collected from rats by puncturing retroorbital sinus. Without delay, glucose solution (50%) was administered through gastric gavage at a dose of 1 g/kg body weight. Three more blood samples were collected at 30, 60, and 120 minutes after glucose administration. All blood samples were collected in 2 mL centrifuge tubes furnished with sodium fluoride and kept on ice until centrifugation. All samples were centrifuged at 3500 ×g for 30 min and the separated plasma specimens were frozen at −80°C until analysis of glucose and insulin.

### 2.5. Insulin Sensitivity Parameters

Plasma glucose was measured by enzyme-based kit method (Biosystems, Barcelona, Spain) and insulin by radioimmunoassay (BARC, India). Insulin sensitivity was measured by Homeostasis Model Assessment of Insulin Resistance (HOMA-IR) and by oral glucose tolerance test as previously reported [21].

### 2.6. Oil Red O Staining

Frozen sections (5 *μ*m) of liver were fixed with 10% formalin for 5 min and stained with oil red O [22]. Images (400x magnification) were taken with Nikon eclipse E800 microscope (Nikon Corporation, Tokyo, Japan) and analyzed with Image-Pro Plus software (Media Cybernetics, Bethesda, USA).

### 2.7. Body Composition Analysis

Body composition was analyzed by Total Body Electrical Conductivity (TOBEC) using small animal body composition analysis system (EM-SCAN, Model SA-3000 Multi detector, Springfield, USA). Lean body mass (LBM), fat-free mass (FFM), and total fat content were calculated from TOBEC according to manufacturer's instructions.

### 2.8. Statistics

Data are reported as mean ± SEM. Data were analyzed by Student's *t*-test by comparing between control and experimental groups at each time point using SPSS software (version 16.0). Data were also analyzed by two-way analysis of variance (ANOVA) to assess the effect of diet, time, and diet over time on the selected parameters. Differences were considered as statistically significant at *p* < 0.05.

## 3. Results

### 3.1. Hepatic and Plasma Lipids

**Figure 1:**
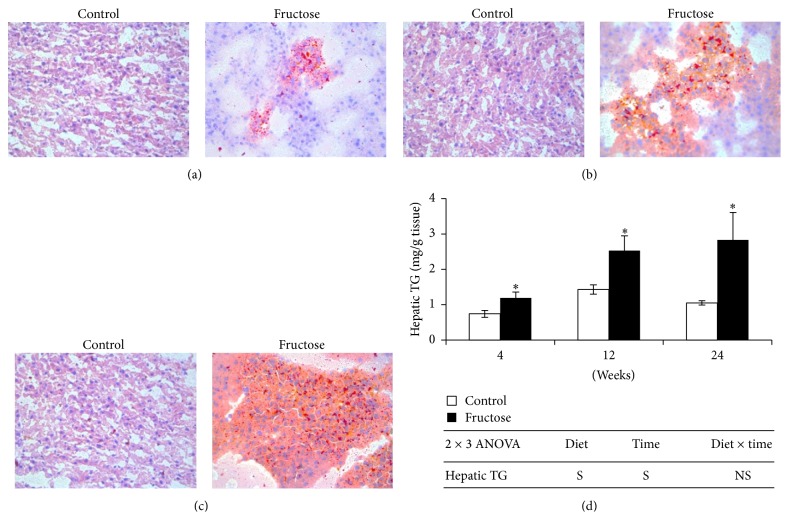
Oil red O staining of control and fructose diet-fed rat livers (400x magnification). (a) 4 weeks. (b) 12 weeks. (c) 24 weeks. (d) Hepatic lipids. Values are in mean ± SE of 8 animals from each group at all time points. ^**∗**^Significance at *p* ≤ 0.05 by Student's *t*-test. Statistical significance of diet, time, and diet *∗* time on individual parameters is also analyzed by two-way ANOVA. “S” indicates “significant” (*p* ≤ 0.05); “NS” indicates “not significant.”

Fructose-feeding for 4, 12, and 24 weeks significantly increased hepatic TG content by 60.8%, 76.9%, and 169.5%, respectively, when compared to that of starch-fed control rats at respective time points (Figure 1(d)). Oil red O staining further confirmed the increase in hepatic lipid content in fructose-fed rats compared with that of control rats (Figures 1(a), 1(b), and 1(c)). We observed microvascular steatosis at 4 weeks of fructose-feeding, whereas both microvascular and macrovascular steatoses were observed at 12 weeks. At 24 weeks, fructose diet-fed rat livers showed majorly macrovascular steatosis along with less microvascular steatosis. Although hepatic lipid content was increased after 4 weeks of fructose-feeding, significant increase in plasma TG level was observed only at 12 weeks, which further continued till 24 weeks (Table 1). A time-dependent increase in hepatic lipid content was observed in fructose-fed rats, whereas no such trend was observed in plasma TG level (Figure 1 and Table 1). Plasma total cholesterol was increased significantly after 12 weeks of fructose-feeding when compared to their respective control rats, whereas no such change was observed at 4 and 24 weeks of fructose-feeding (Table 1). Plasma HDL cholesterol levels were not affected by fructose-feeding at all three time points (Table 1).

### 3.2. Insulin Sensitivity and Glucose Tolerance

**Figure 2:**
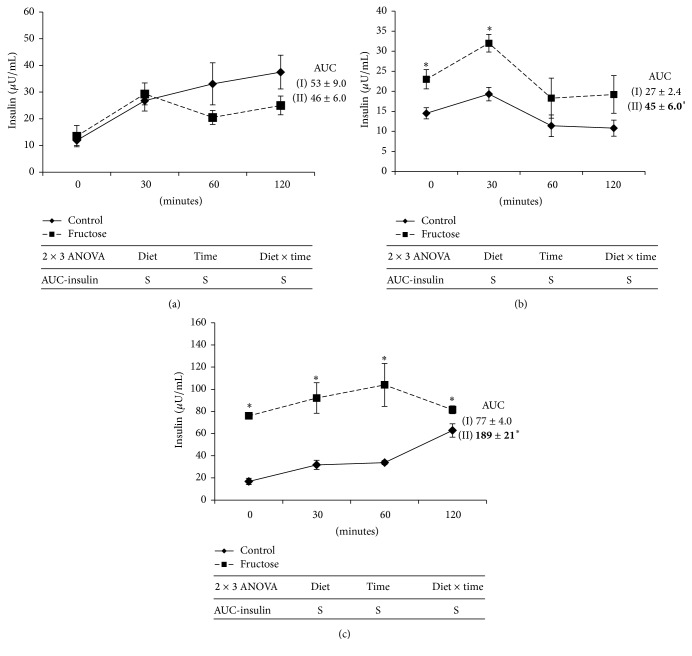
Plasma insulin responses to an oral glucose load in control and fructose diet-fed rats. (a) 4 weeks. (b) 12 weeks. (c) 24 weeks. (—) Control group. (- - - -) High-fructose-fed group. Values are in mean ± SE of 8 animals from each group at all time points. ^**∗**^Significance at *p* ≤ 0.05 by Student's *t*-test. Statistical significance of diet, time, and diet *∗* time on individual parameters is also analyzed by two-way ANOVA. “S” indicates “significant” (*p* ≤ 0.05); “NS” indicates “not significant.”

Fasting plasma insulin level was not altered by 4 weeks of fructose-feeding, whereas it was increased by 64.2% and 347% at 12 and 24 weeks of fructose-feeding, respectively, when compared to that of respective control rats (Table 1). Insulin sensitivity measured from HOMA-IR and insulin AUC during oral glucose tolerance test (OGTT) showed similar trend as observed in fasting plasma insulin levels in fructose-fed rats when compared to that of respective starch-fed rats (Table 1 and Figure 2). Fasting blood glucose was not altered by fructose-feeding at all time points, whereas glucose tolerance measured from glucose AUC during OGTT also showed similar trend except at 4-week time point, where it showed marginal but significant increase when compared to that of respective control group rats (Table 1).

### 3.3. Tissue Weights, Food Intake, and Body Composition

**Figure 3:**
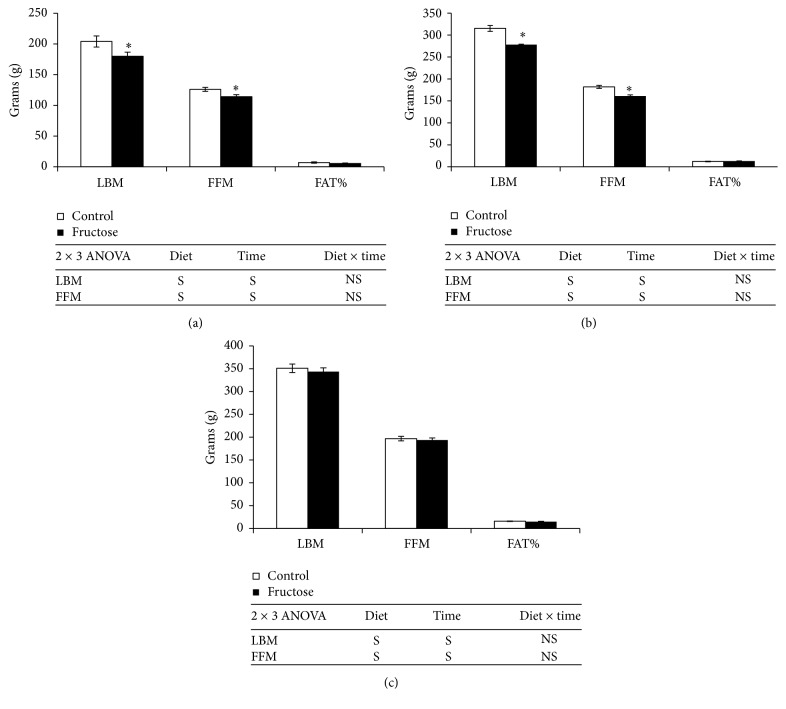
Body composition of control and fructose diet-fed rats. (a) 4 weeks. (b) 12 weeks. (c) 24 weeks. Values are in mean ± SE of 8 animals from each group at three time points. ^**∗**^Significance at *p* ≤ 0.05 by Student's *t*-test. Statistical significance of diet, time, and diet *∗* time on individual parameters is also analyzed by two-way ANOVA. “S” indicates “significant” (*p* ≤ 0.05); “NS” indicates “not significant.”

A significant decrease in testis weight (33%) was observed after 4 weeks of fructose-feeding in experimental rats compared to that of respective control rats, but weights were similar between the groups after 12 and 24 weeks of fructose-feeding (Table 2). Kidney weights were significantly increased at 24 weeks (19%, resp.) of fructose-feeding in experimental rats when compared to those of respective starch-fed control rats (Table 2). Food intake was not affected by fructose-feeding at all the time points (Table 2). Although hepatic TG levels were increased significantly after 4 weeks of fructose-feeding, increase in liver weight was observed only after 12 weeks of fructose-feeding, which was further exacerbated at 24 weeks in fructose-fed-rats when compared to their respective control rats (Table 2). Lean body mass (LBM) and fat-free mass (FFM) were significantly decreased after 4 and 12 weeks of fructose-feeding, which were normalized after 24 weeks when compared with those of respective control rats (Figure 3). Epididymal fat depot weights were significantly decreased after 4 weeks of fructose-feeding in experimental rats compared with those of respective control rats; however, the weights were comparable with control rats after 12 and 24 weeks of fructose-feeding (Table 2).

### 3.4. Plasma Testosterone and Homocysteine

**Figure 4:**
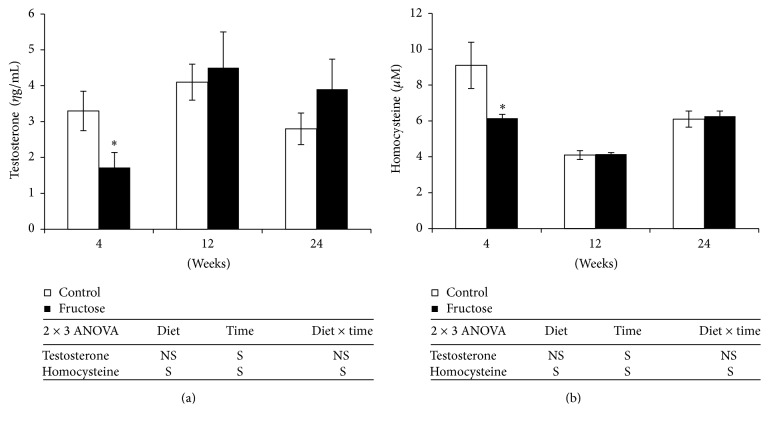
(a) Plasma total testosterone levels. (b) Homocysteine levels after 4, 12, and 24 weeks of control and fructose diets feeding in Sprague Dawley rats. Values are in mean ± SE. ^**∗**^Significance at *p* ≤ 0.05 by Student's *t*-test. Statistical significance of diet, time, and diet *∗* time on individual parameters is also analyzed by two-way ANOVA. “S” indicates “significant” (*p* ≤ 0.05); “NS” indicates “not significant.”

In line with decrease in testis weight, plasma testosterone levels were decreased significantly by 47.9% after 4 weeks of fructose-feeding when compared to those of control rats, whereas they reached normal levels after 12 weeks of fructose-feeding which continued till 24 weeks (Figure 4(a)). Plasma homocysteine levels also showed similar trend as that of testosterone, which were decreased significantly by 34.5% after 4 weeks of fructose-feeding (Figure 4(b)).

## 4. Discussion

In the present study, we explored the sequential development of metabolic syndrome features including dyslipidemia, insulin resistance, and visceral obesity in fructose-fed SD rat model. We also reported the variations of circulatory testosterone and homocysteine during the course of MS development. Hepatic and circulatory TG levels and insulin resistance were increased during the course of fructose-induced MS development but not the visceral obesity with increased hepatic TG content as an early metabolic disturbance. Interestingly, decreased circulatory testosterone and homocysteine levels are the initial metabolic signatures before the development of dyslipidemia and IR in this model. According to our knowledge, this is the first study to report the transient decrease of circulatory testosterone and homocysteine before the onset of metabolic syndrome features in fructose-fed rat model.

Liver is the major site for fructose metabolism [23]. Fructose is considered to be highly lipogenic metabolite compared to glucose, as its metabolism in liver bypasses two key regulatory glycolytic reactions catalyzed by glucokinase and phosphofructokinase [23]. Thereby fructose generates excessive acetyl Coenzyme-A (acetyl-CoA) and glyceraldehyde-3-phosphate, two key precursors for biosynthesis of triglycerides. It also increases hepatic TG accumulation by increasing expression of lipogenic genes through inducing nuclear translocation of carbohydrate response element-binding protein (ChREBP) transcription factor [24]. ChREBP is also proposed to downregulate peroxisome proliferator activated-receptor *α* (PPAR*α*), an essential transcription factor for expression of fatty acid *β*-oxidation enzymes [25]. Masterjohn et al. reported that 9 weeks of fructose-feeding to SD rats significantly increases liver weight and plasma TGs, but interestingly there was no change in hepatic TG content [26]. In contrast to this study, 4 weeks of fructose-feeding resulted in increased hepatic TG levels in our study. In fact, it is the initial event in the development of metabolic syndrome in this model ahead of other risk factors, and possibly it might have played critical role in development of MS risk factors at later stages.

Plasma TG levels are mainly regulated by hepatic TG synthesis, release, and peripheral tissue utilization. Studies on human subjects as well as in animal models showed that fructose-feeding increases the plasma TG levels. Stanhope et al. reported that fructose-feeding (25% of energy consumption) in human subjects increased area under the curve of plasma TG [6]. Same study reported that fructose-feeding for 24 hrs is enough to increase circulatory TGs in human subjects [6]. In SD rats, fructose-feeding was shown to increase plasma TG as early as two weeks, whereas other studies reported the similar observation between 3-week duration and 16-week duration [27–32]. In contrast to the studies that reported early increase (2 to 4 weeks) in plasma TG, in our study, we have not observed increase in plasma TGs after 4 weeks of fructose-feeding, even though we observed increased hepatic TG content. We observed the increased plasma TGs after 12 and 24 weeks of fructose-feeding. Possibly differences in fructose content of the diets (in between 55 and 65%) and age of rats and also different fasting durations might have been responsible for the different observations in plasma TG after fructose-feeding in SD rats. A recent study also reported that older rats respond to fructose-induced dyslipidemia better than the younger rats, although in different rat strain (Fischer rats) [33]. These observations strongly support the previous studies that reported hypertriglyceridemia as an important MS risk factor developed by fructose-feeding in this rat model.

Although hypertriglyceridemia is one of the major metabolic syndrome factors observed in fructose-fed rat model, there are no such strong observations reported with respect to hypercholesterolemia. A recent meta-analysis of human studies reported that fructose intake is positively correlated with total cholesterol levels when the daily intake of fructose is more than 100 g per day but not when the intake is less than that, suggesting the critical role of fructose quantity in regulating plasma cholesterol levels [34]. Majority of the studies on SD rats reported no change in plasma total cholesterol levels after fructose-feeding [26, 28], whereas Chou et al. showed increased levels after four weeks of fructose-feeding [31]. In contrast to this report, we have not observed any increased total cholesterol after 4 weeks of fructose-feeding, but we observed a small increase in plasma total cholesterol levels at 12 weeks of fructose-feeding but they reached normal levels after 24 weeks.

Insulin resistance is another important MS feature developed by fructose-feeding in rats. Silbernagel et al. reported decreased insulin sensitivity in healthy adults after fructose-feeding (150 g/day) for 4 weeks [7]. Fructose-feeding decreases insulin receptor substrate-2 (IRS-2) expression in liver [35]. Majority of the studies reported that tissues and systemic TG levels play critical role in fructose-induced insulin resistance. Studies showed that hepatic TG content positively correlates with hepatic insulin resistance and reduction of hepatic TG ameliorates hepatic insulin resistance [36]. Liver and skeletal muscle TG increases insulin resistance majorly by diacylglycerol-mediated activation of protein kinase C (PKC) which impairs insulin signaling [37]. Increased hepatic insulin resistance also increases insulin release from pancreas as a compensatory mechanism. Although in the present study we observed increased hepatic TG after 4 weeks of fructose-feeding, we have not observed elevation of fasting plasma insulin levels or insulin AUC during OGTT. We observed elevated fasting insulin levels, HOMA-IR, and increased AUC only after 12 weeks of fructose-feeding, which worsened after 24 weeks, suggesting gradual increase in systemic insulin resistance with fructose-feeding. As reported by previous studies, initial increase in hepatic TG after 4 weeks of fructose-feeding might have resulted in hepatic insulin resistance and resulted in increased insulin secretion which is evidenced after 12 weeks of feeding. Development of insulin resistance in tissues like skeletal muscle might have further worsened insulin resistance after 24 weeks of fructose-feeding. Although some of the studies reported role of visceral obesity in fructose-induced insulin resistance, in our study, as we have not observed visceral obesity, contribution of it to the observed insulin resistance in our study might be negligible. Korandji et al. [27] reported feeding of 60–65% fructose diet increases fasting plasma glucose after one week, whereas Padiya et al. [28] and Chou et al. [31] reported the similar observation after 4 to 8 weeks of fructose-feeding. Masterjohn et al. [26] and Leibowitz et al. [32] reported no effect of fructose-feeding on glucose levels after 5 to 9 weeks of feeding. In line with observations of Masterjohn et al. and Leibowitz et al., we have not observed any change in fasting plasma glucose levels even after 24 weeks of fructose-feeding. Leibowitz et al. [32] reported no change in fasting plasma insulin levels after fructose-feeding for 5 weeks, whereas Chou et al. [31] reported increased plasma insulin after 4 weeks of fructose-feeding. In support to observations of Leibowitz et al. [32], in our study, we have not observed increase in fasting plasma insulin level or insulin AUC after 4 weeks of fructose-feeding but we observed elevated fasting insulin and insulin AUC at 12 weeks of fructose-feeding which worsened after 24 weeks.

Recent studies reported that fructose induces preadipocyte differentiation [38]. Role of dietary fructose in the development of human obesity is not well understood as clinical studies reported contrasting observations [39]. Our earlier studies [40, 41] and D'Alessandro et al. [42] reported that sucrose feeding increases adiposity in Wistar rats. However, in the present study, we did not observe increase in adiposity by fructose-feeding which could be possibly due to strain variation. Majority of studies on fructose-induced MS in SD rats reported no effect of fructose on body weight and visceral obesity [28, 31, 32]. In support to these studies, we have not observed increase in visceral fat by fructose-feeding for 12 and 24 weeks; instead, we observed a transient decrease in visceral fat depots at earlier phase (4 weeks) which was also reported by Masterjohn et al. [26] and Korandji et al. [27]. Based on our results, we propose that SD rat model is a good model to study fructose-induced MS independent of visceral obesity.

Apart from mediating male reproductive functions, testosterone was shown to be pathologically involved in the development of type 2 diabetes and CVDs. Clinical and animal studies reported the possible role of testosterone in the development of MS components including dyslipidemia, IR, and visceral obesity [9–11]. In human subjects, circulatory testosterone levels were negatively correlated with visceral obesity, IR, and fatty liver [9–11]. Similar observations were also reported in animal models [43] and testosterone replacement ameliorated MS symptoms [9–11]. El Hafidi et al. reported low levels of testosterone in sucrose-fed rats for 20 weeks and no other study has noticed that in high-fructose-fed rats [44]. In our study, fructose-feeding for 4 weeks reduced testosterone levels which were normalized after 12 and 24 weeks of feeding. Skeletal muscle mass and plasma homocysteine levels also followed the same trend as that of testosterone, except that reduced skeletal muscle mass normalized after 24 weeks of fructose-feeding. As testosterone promotes the differentiation of satellite cells to myocytes, the decreased muscle mass after 4 weeks of fructose-feeding was expected in our study. Skeletal muscle reduction even after testosterone normalization after 12 weeks of fructose-feeding is possibly due to characteristic long-term action of steroid hormones. Decreased testosterone levels were also associated with lowered homocysteine levels after 4 weeks of fructose-feeding in our study. Previous studies reported that testosterone increases circulatory homocysteine levels by decreasing expression of cystathionine *β*-synthase (CBS), which is involved in conversion of homocysteine to cystathionine and this was proposed to be one of the possible mechanisms for the higher homocysteine levels observed in male subjects [45]. As testosterone deficiency was shown to induce hepatic steatosis by regulating expression of lipolytic and lipogenic genes, possibly the observed testosterone deficiency might be contributed to the observed fatty liver in addition to lipogenic metabolism of fructose in our study [46]. Although testosterone levels were normalized after 12 and 24 weeks of fructose-feeding, hepatic TGs and insulin resistance were exacerbated. It may be possible that lower testosterone levels during early phase of fructose-feeding might have epigenetically programmed the development of MS at later stages. The mechanisms by which dietary fructose induces testosterone deficiency will be interesting for future investigations. The decreased circulatory testosterone levels are possibly due to impairment of testicular development observed after 4 weeks of fructose-feeding in this study. Studies reported that high glucose levels are essential for testicular development [47]. Possibly altered glucose metabolism due to fructose-feeding might have impaired the testis development at early stages. In support to our observation, a recently published short report during our experiment reported a significant decrease in testis weight in weaning Wistar rats which were fed with 70–80% fructose diets and the authors also reported that glucose supplementation restored the weight of testis [48].

Homocysteine levels were shown to be elevated in MS in human subjects [17] and to associate with insulin resistance, fatty liver, and obesity [14–16]. Interestingly, Rosolová et al. [49] reported that plasma homocysteine is negatively correlated with insulin resistance in healthy subjects and also hypohomocysteinemia was observed in initial phases in type 2 diabetic subjects [50]. El Masellamy et al. [18] reported that fructose-feeding for 5 weeks to Wistar rats increased homocysteine along with features of MS. In contrast to this study, we observed decreased plasma homocysteine levels after 4 weeks of fructose-feeding which were normalized at later stages. As circulatory homocysteine levels can alter global DNA methylation [51], role of lowered Hcy levels in epigenetic programming of MS cannot be ruled out in this model.

## 5. Conclusions

Here we conclude that transient decrease in circulatory testosterone and homocysteine levels and increased hepatic TG content are the earliest metabolic disturbances followed by hypertriglyceridemia and insulin resistance in fructose-fed SD rats. Further studies in this model may give more insights into role of testosterone and homocysteine in the development of fructose-induced MS.

## Figures and Tables

**Table 1:** Plasma parameters.

	4 weeks		12 weeks		24 weeks	
	Control	Fructose	Control	Fructose	Control	Fructose
Plasma glucose(mg/dL)	70 ± 2.4	79 ± 3.6	79 ± 1.6	84 ± 1.9	88 ± 1.3	76 ± 3.0
Plasma insulin (*µ*U/mL)	12 ± 2.0	13 ± 4.0	14 ± 1.4	23 ± 2.4^*∗*^	17 ± 3.0	76 ± 19^*∗*^
AUC-glucose (mg/dL·min)	188 ± 4.6	203 ± 4.3^*∗*^	219 ± 6.6	228 ± 6.6	237 ± 9.5	220 ± 5.7
HOMA-IR	2 ± 0.34	3 ± 0.85	3 ± 0.31	5 ± 0.5^*∗*^	4 ± 0.6	18 ± 4.0^*∗*^
Plasma TG (mg/dL)	42 ± 4.0	47.3 ± 5.0	37 ± 2.4	52 ± 6.0^*∗*^	40 ± 1.9	54 ± 4.1^*∗*^
Cholesterol (mg/dL)	82 ± 4.0	82 ± 6.1	72 ± 2.2	89 ± 6.4^*∗*^	77 ± 3.08	79 ± 4.0
HDL (mg/dL)	67 ± 6.6	67 ± 4.1	56 ± 2.5	67 ± 5.2	68 ± 2.92	69 ± 3.0

2 × 3 ANOVA	Diet	Time	Diet × Time			

Plasma insulin (*µ*U/mL)	S	S	S			
HOMA-IR	S	S	S			
Plasma triglycerides	S	NS	NS			

Values are in mean ± SE of 8 animals from each group at three time points. “*∗*” indicates statistical significance (*p* ≤ 0.05) of control and fructose-fed groups at each time point analyzed by Student's *t*-test. Statistical significance of diet, time, and diet *∗* time on individual parameters is also analyzed by two-way ANOVA. “S” indicates “significant” (*p* ≤ 0.05); “NS” indicates “not significant.”

**Table 2:** Physical parameters.

	4 weeks		12 weeks		24 weeks	
	Control	Fructose	Control	Fructose	Control	Fructose
Final body weight (g)	242 ± 9.0	208 ± 7.0^*∗*^	372 ± 9.0	332 ± 13.0^*∗*^	430 ± 11	418 ± 10
Body weight gain (g)	169 ± 9.0	136 ± 6.4^*∗*^	302 ± 8.5	260 ± 11.0^*∗*^	360 ± 10	348 ± 9.0
Daily food intake (g)	14 ± 0.40	13 ± 0.40	16 ± 0.30	15 ± 0.40	16 ± 0.20	16 ± 0.30
Liver (g)	7 ± 0.25	8 ± 0.29	8 ± 0.31	10 ± 0.41^*∗*^	10 ± 0.40	12 ± 0.40^*∗*^
Retroperitoneal fat (g)	2 ± 0.20	1 ± 0.18	3 ± 0.30	4 ± 0.56	6 ± 0.70	6 ± 0.70
Epididymal fat (g)	3 ± 0.31	2 ± 0.3^*∗*^	5 ± 0.41	5 ± 0.44	7 ± 0.70	6 ± 0.70
Testis (g)	3 ± 0.14	2 ± 0.08^*∗*^	3 ± 0.07	3 ± 0.05	3 ± 0.08	3 ± 0.18
Kidney (g)	2 ± 0.05	2 ± 0.06	2 ± 0.18	3 ± 0.07	2.6 ± 0.08	3.1 ± 0.05^*∗*^
Pancreas (g)	1.8 ± 0.06	1.7 ± 0.06	1.0 ± 0.03	1.0 ± 0.07	0.4 ± 0.10	0.5 ± 0.05

2 × 3 ANOVA	Diet	Time	Diet × time			

Final body weight	S	S	NS			
Body weight gain	S	S	NS			
Liver	S	S	NS			
Kidney	S	S	S			

Values are in mean ± SE of 8 animals from each group at three time points. “*∗*” indicates statistical significance (*p* ≤ 0.05) of control and fructose-fed groups at each time point analyzed by Student's *t*-test. Statistical significance of diet, time, and diet *∗* time on individual parameters is also analyzed by two-way ANOVA. “S” indicates “significant” (*p* ≤ 0.05); “NS” indicates “not significant.”

## Abbreviations

AUC: Area under the curve
CBS: Cystathionine *β*-synthase
ChREBP: Carbohydrate response element-binding protein
CVD: Cardiovascular disease
FFM: Fat-free mass
Hcy: Homocysteine
HDL: High-density lipoprotein
HOMA-IR: Homeostasis Model Assessment of Insulin Resistance
IR: Insulin resistance
IRS: Insulin receptor substrate
LBM: Lean body mass
MS: Metabolic syndrome
OGTT: Oral glucose tolerance test
PKC: Protein kinase C
PPAR*α*: Peroxisome proliferator activated-receptor *α*
SD rat: Sprague Dawley rat
TG: Triglyceride.
